# Effects of Short Video Addiction on the Motivation and Well-Being of Chinese Vocational College Students

**DOI:** 10.3389/fpubh.2022.847672

**Published:** 2022-05-10

**Authors:** Jian-Hong Ye, Yu-Tai Wu, Yu-Feng Wu, Mei-Yen Chen, Jhen-Ni Ye

**Affiliations:** ^1^Faculty of Education, Beijing Normal University, Beijing, China; ^2^Office of Physical Education, Soochow University, Taipei City, Taiwan; ^3^Office of Physical Education, Ming Chi University of Technology, New Taipei City, Taiwan; ^4^Graduate Institute of Sport, Leisure and Hospitality Management, National Taiwan Normal University, Taipei City, Taiwan; ^5^Graduate Institute of Technological and Vocational Education, National Taipei University of Technology, Taipei City, Taiwan

**Keywords:** micro ecological system theory, flow experience, learning motivation, learning well-being, short video addiction, short video flow, person-process-content

## Abstract

While media use can be beneficial in some ways, excessive use of media has led to growing concerns about its potential negative consequences. With the popularity of Chinese video applications (apps) such as DouYin, TikTok, Kwai, and other short video apps sweeping through schools around the world. Due to the diversity and immersion principle of short videos, their popularity continues to grow, and the phenomenon of students being addicted to short videos also brings many hidden dangers to the learning effect. Among other things, the problem of excessive use of the Internet among Chinese youth has led the government to propose a series of control policies to strengthen the monitoring of harmful habits of youth in the use of online applications. In addition, the problem of youth addiction to short videos has become a major concern for education experts and the general public, thus demonstrating that short video addiction is indeed an ongoing research issue. Therefore, this study aimed to understand the causes of short-form video addiction and its impact on the psychology of learning, and to investigate the relationship between short-form video flow experience, short-form video addiction, intrinsic and extrinsic learning motivation, and learning well-being from the perspectives of flow experience theory and micro ecological systems. The questionnaire was sent *via* instant messaging software such as QQ and WeChat, and university students from vocational colleges in China were invited to complete the questionnaire. A total of 517 valid data were collected, including 222 (42.9%) were male students and 295 (57.1%) were female students. The collected questionnaires were analyzed for reliability and validity after removing incomplete data, followed by structural equation modeling for model verification. The findings showed that: (1) short video flow experience had a positive effect on short video addiction; (2) short video addiction had a negative effect on intrinsic and extrinsic learning motivation; (3) intrinsic and extrinsic learning motivation had a positive effect on learning well-being; (4) short video flow experience had an indirect negative effect on intrinsic and extrinsic learning motivation; (5) short video flow and short video addiction had indirect negative effects on learning well-being. According to the results, it is clear that addiction to short videos has a negative impact on learners' learning motivation and positive psychology of learning, so parents and teachers should effectively guide students to use short video apps in a self-controlled way.

## Introduction

In the past two decades, new technologies and the Internet have undoubtedly changed our lives; this includes how we deal with the many great advantages brought by the Internet and the dangers that may be caused by abuse of the Internet (1). The use of social media is common and essential in many people's lives (2). Although media use is beneficial in some ways, excessive media use has led to growing concerns about its potential negative consequences, such as addiction (3). Some young people face problems using these technologies or the risk of becoming addicted to them (4). Therefore, Internet-related diseases have aroused increasing research interest, and further investigation is needed (5).

Internet addiction can be divided into two types of definitions, broad Internet addiction, which encompasses general and multidimensional Internet overuse behaviors, and specific Internet addiction, which has similar characteristics to general Internet addiction, which refers to things like smartphone addiction, social media addiction, etc. (6). The short- video addiction that is the subject of this study is a specific type of Internet addiction. Therefore, Leung and Chen (3) suggested that future research on Internet addiction should focus on addiction to specific behaviors or types of content, especially because different applications provide a large amount of content on the Internet that can be accessed through mobile devices.

In the field of Internet addiction research, short videos are an independent and emerging Research Topic with a clear definition of terms, unlike social media, which is such a large border area. Short videos are videos that are <15 min in length, mostly between 1 and 5 min, and have a clear theme type. Because short videos are characterized by a clear style, concise content, and fast rhythm, most of the video content will attract users' attention within seconds and make them keep watching (7), which also causes users to become silently immersed, and can gradually create addictive behaviors. Due to the diversity and immersion principle of short videos, their popularity continues to be unabated, and the phenomenon of students being addicted to short videos brings many hidden dangers for the effects of learning. However, there is still little discussion on the negative impact of short video addiction in the learning psychology literature. Therefore, this study examined the impact of short video addiction on learning in order to expand the understanding of the impact of short videos.

Although there are still few studies on short video addiction, Internet addiction is a new form of dependence on connected devices, and such addictive behaviors can cause psychological problems and leave many negative effects. For example, Chen et al. (6) study found that psychological distress was associated with the degree of Internet use. Therefore, from the perspective of positive psychology, addiction would be in conflict with positive psychology. Positive psychology originated in the second half of the 20th century with a call for greater emphasis on the positive aspects of life (8). In other words, from the perspective of positive psychology, it is important to avoid or exclude factors that have a negative impact on life. Similarly, negative factors that affect school life are also excluded.

Previous research has demonstrated that Internet addiction can have a negative impact on the psychological and emotional development of adolescents (9), which means that it may also affect learners' perceptions of well-being while learning, which is a rather undesirable situation for learning. This means that happiness is not only the disappearance of negative emotions and emotional states, but also the existence of positive emotions and emotional states (10).

In order to systematically understand the causes of short video addiction and its effects on learners, this study was conducted based on the micro ecological system to consider the trend of youth mental health education from an ecosystem perspective (11). Brofenbrenner's (12) micro ecological system was proposed to explore the interaction between person-process-content (PPC), where ecology refers to the degree of fit between an individual and the environment, not only to promote the development of the environment, but also to provide an environment for the survival of the individual, according to which there is a close connection between the individual and the environment. That is to say, the interaction between scholars and short videos may have a close influence on the learning environment.

In summary, although short video addiction has received much attention and heated discussion from the public, there is still a lack of related study on the topic of short video addiction. Therefore, this study used the ecosystem theory to construct a research model to investigate the relationship between short video flow, addiction, intrinsic and extrinsic learning motivation, and learning well-being among Chinese vocational school students, in the hope of shedding light on the prevention and education of addictive behaviors at home or on campus.

## Research Model and Hypotheses

### Research Model

Bronfenbrenner (12) proposed that the interactions between PPC can be explored from the perspective of micro ecological systems. While, Hong et al. (13) further found that PPC can be used to explore the PPC interaction in educational settings, especially in the context of Internet-based addictive behaviors. In addition, Gabbard and Krebs (14) explained that in PPC, “process” refers to the integration and interaction between the individual and the content; “person” refers to the individual's cognition and emotion; and “content” refers to the objects and environment of interaction.

**Figure 1:**
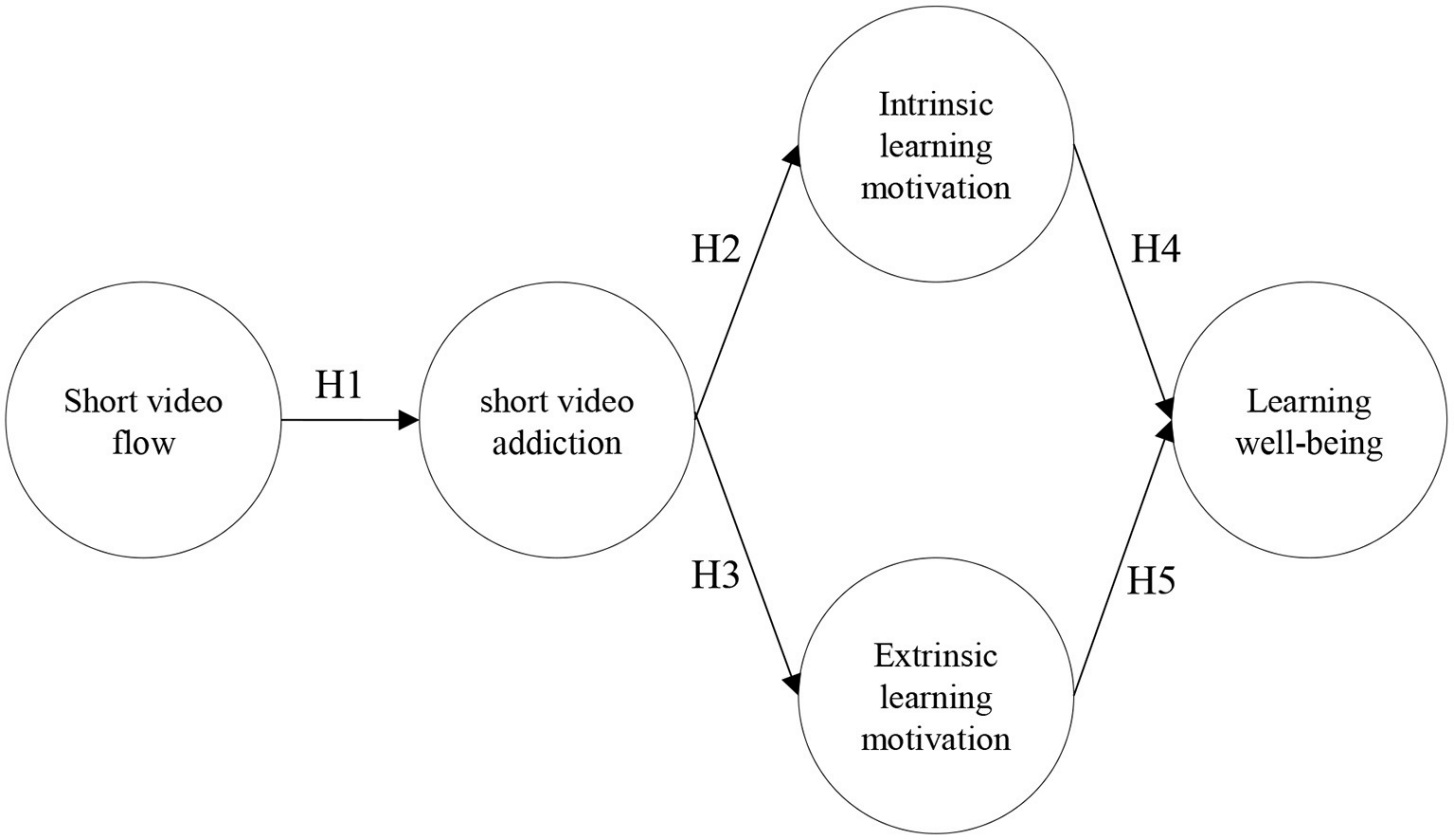
Research model.

This study considers “person” as the perception of flow experience when watching short videos, “content” as the addictive nature of interaction with the cell phone, and “process” as the learner's motivation to learn, and the perceived subjective well-being in their learning life. That is, when learners often experience high levels of immersion in the process of watching short videos, they will begin to develop video dependency, which will weaken their motivation to learn and affect their perceived positive feelings about their learning lives. Accordingly, it is worth exploring whether there is a relationship between short video flow and the control of cell phone addiction among technical high school students. Therefore, Therefore, this study explored from the perspective of integrating flow experience and ecosystem theories to understand the relationship between short video flow, short video addiction, intrinsic and extrinsic learning motivation, and learning well-being, as shown in Figure 1.

### Research Hypotheses

#### Short Video Flow and Short Video Addiction

Flow is defined as the experiences that a person immerses him/herself in to be fully involved in the process of an activity (15). In recent years, with the rise in popularity of short videos, flow experience has been commonly used in studies related to human-computer interaction (16). Several studies have implied that flow experience is related to media addiction (e.g., online addiction, Internet addiction, video addiction) (17, 18). Some researchers have argued that addiction can be derived from positively reinforcing psychological processes (7), which seems to be particularly important for the relations between flow and addictive media use (19). That is, when people experience high levels of flow experience while watching short videos, they are more likely to develop addiction problems. Therefore, the following hypothesis was proposed:

H1: Short video flow has a positive effect on short video addiction.

#### Short Video Flow and Learning Motivation

Internet has brought easy access to short videos, which are potentially easy for individuals to become addicted to. Short video addiction is defined as the excessive and irrational use of short videos to a point where it influences one's daily life (20, 21). Several studies have shown that addiction can have a negative effect on emotional reactions, mood, and psychological problems (22, 23). Most recently, it has been found that addiction can cause negative learning motivations in children (24). That is, when people have a high level of video addiction, it will have a negative impact on their learning motivation. Therefore, students with good learning motivation will potentially have better learning processes and results when they do not have other things on their mind (24) such as short videos. Thus, the following hypotheses were proposed:

H2: Short video addiction has a negative effect on intrinsic learning motivation.

H3: Short video addiction has a negative effect on extrinsic learning motivation.

#### Learning Motivation and Learning Well-Being

Motivation is an important determinant of students' learning success rate (25). Therefore, the development of motivation is essential for individuals to have the ability to cope with new environments (26). One study has suggested that motivation is a main factor of how much information students absorb from the learning materials presented to them or how much they learn in a learning activity (27). In the Education field, motivation for learning is considered a very important factor, and it is a force that pushes students toward a better learning process and helps them achieve their academic responsibilities (26). Motivation can be seen from two perspectives; intrinsic motivation is defined as learning due to interest or enjoyment of learning. In contrast, extrinsic motivation refers to learning because of certain ends, such as being in a higher position in class and getting better grades (28).

Furthermore, a recent study provided evidence that motivation is important for student well-being (27), while other studies have also shown that Chinese students' motivation is an important predictor of their academic success and well-being (29, 30). Many elements have proven valuable to well-being in youth and adults, while in the field of education, well-being has been conceptualized as both an outcome and a process (31). That is, the more positive motivation people have for learning, the better they feel about their subjective well-being in their learning lives. Therefore, the following hypotheses were proposed:

H4: Intrinsic learning motivation for learning has a positive effect on learning well-being.

H5: Extrinsic learning motivation has a positive effect on learning well-being.

#### Short Video Flow and Learning Motivation

Students' learning motivation to enjoy and continue learning is a critical reason for academic success. Motivation can be defined as intrinsic and extrinsic, where intrinsic motivation means being able to enjoy or engage in an activity without any reinforcement, whereas extrinsic motivation requires outcomes such as achievement incentives (32). However, when in flow, people are highly focused and concentrate on what they are doing at a given moment (33). In line with this definition, people who are in a state of short video flow will have lower learning motivation. Therefore, the following hypotheses were proposed:

H6: Short video flow has an indirect negative effect on intrinsic learning motivation.

H7: Short video flow has an indirect negative effect on extrinsic learning motivation.

#### Short Video Flow and Learning Well-Being

Although there is a great deal of research on Internet flow, the impact of short video flow on learning is still unknown. However, some clues can be deduced from other studies. For example, from the perspective of psychological symptoms, it has been observed that when playing games, addicts present a highly entertaining state where they cannot stop their activities, desire more time to play games, and feel empty, depressed, and irritable when they are not playing, with all the harmful consequences that these symptoms have on the individual's life (34). According to these concepts, it will also be harmful to their academic life. What Griffiths describes is an addict who goes through a highly enjoyable flow experience and is unwilling to leave the addiction, and the negative impact it has on their life. In addition, Laffan et al. (35), who explored online game participation and players' overall well-being, found a negative and weak relationship between online game flow and overall well-being. That is, when students are highly immersed in short videos, it may have a negative impact on their personal well-being. Therefore, based on the above literature, the following hypotheses were proposed:

H8: Short video flow has an indirect negative effect on learning well-being.

#### Short Video Addiction and Learning Well-Being

Well-being also refers to an individual's quality of life, is an important factor that predicts individuals' natural and social environments (36), and can be affected by behavioral addiction (5). Past studies have confirmed that addiction may negatively affect users' well-being (7). However, recent studies have shown that the intensity of online activity is negatively associated with subjective well-being (37). Teenagers' psychological health is affected by the risk of Internet addiction (1). Internet addiction has been found to be widespread worldwide, including in Asian countries, and is associated with a number of negative outcomes and other behavioral addictions (5). Therefore, the following hypothesis was proposed:

H9: Short video addiction has an indirect negative effect on learning well-being.

## Research Method

### Procedure

This study used a descriptive and relevant cross-sectional method, and the snowball sampling method when issuing the questionnaire. This way, the questionnaire link is sent to the communication group of university students in vocational schools in China in instant messaging software such as QQ and WeChat. After inviting them to fill out the questionnaire and forward it to their peers, the questionnaire was collected from October 20, 2020. The collection of data was closed after receiving 600 questionnaires. In this study, it was ensured that all processes conducted in the study comprising humans were in accordance with the ethical principles of the American Psychological Association and in compliance with Chinese regulatory requirements. Informed consent was provided in the survey, so that all participants were aware that they were participating in this study and that the data they provided were presented anonymously.

### Participants

In this study, 600 questionnaires were collected, and 83 invalid questionnaires with incomplete answers or that took <3 min to complete were deleted, resulting in 517 valid questionnaires, with a valid recovery rate of 86.2%. Among them, 222 (42.9%) were male students and 295 (57.1%) were female students; 56 (10.8%) watched short videos for 1–3 days per week, 130 (25.1%) watched for 4–6 days per week, and 331 (64%) watched every day. The average viewing time per day was <1 h for 61 participants (11.8%), between 1 and 3 h for 266 participants (51.4%), and more than 3 h for 190 participants (36.8%); the average age of participants was 19.19 years old (the standard deviation was 1.12 years).

### Questionnaire

This was a quantitative study in which data were collected through a questionnaire. The content of the questionnaire was adapted from past research instruments and associated theories. Three revisions were made for the fluency and comprehensibility of the text description of the item, and the completeness of the connotation of the aspect was reviewed by three educational experts for content validity. The content of the questionnaire was rated on a 5-point Likert scale, with 1 indicating *strongly disagree* to 5 indicating *strongly agree*. The greater the assessment score, the greater the tendency to have a high level of that construct.

#### Short Video Flow

Csikszentmihalyi (38) defined flow experience theory. It was proposed that flow experience is an overall assessment and a state of mind in which an individual participates in a certain activity. Based on the above definition, this study referred to and adapted the flow experience scale from Hong et al. (39) with eight questions to measure participants' perceptions of positive experiences of high immersion and enjoyment experienced while watching short videos. Example items are: “When I watch short videos, I keep watching to forget myself” and “When I watch short videos, I always concentrate on them and forget about other things.”

#### Short Video Addiction

Media addiction, as a mental disorder and a complex biopsychosocial phenomenon, is defined as a user's inability to control media use and the adverse effects it has on the user's daily life (3). According to this definition, the present study referred to and adapted the Game Addiction Scale from Wu et al. (40) with eight items to measure the participants' perceived level of self-viewing short videos to addiction. Example items are: “I will put aside what should be done or executed and spend my time watching short videos” and “I get depressed if I don't watch short videos.”

#### Learning Motivation

According to Deci and Ryan (41), an individual's intrinsic motivation is to engage in a behavior in order to gain pleasure and satisfaction from the process of participation, while extrinsic motivation is to engage in an activity in order to obtain an external benefit. Based on the above definition, six questions each on intrinsic and extrinsic learning motivation were designed to measure participants' perceptions of internal and external motivation to participate in the learning process. Example intrinsic learning motivation items are: “I learn so that I can feel the joy of discovering new things” and “I learn so that I can continue to learn about things that interest me.” Example external learning motivation items are: “I am studying so that I can find a more prestigious job in the future” and “I am studying hard so that I can eventually get into a career that I like.”

#### Learning Wellbeing

In well-being research, one of the definitions of well-being is based on subjective evaluations of the quality of life, including positive and negative effects and cognitive evaluations of life satisfaction, which are related to the attainment of happiness (42). Based on the above definition, this study adapted Lu and Lin's (43, 44) “Very Short Version of the Chinese Well-Being Scale” with 10 items to measure participants' perceptions of subjective well-being in academic life. Example items are: “I feel happy in my learning experience” and “I feel a sense of accomplishment in my learning performance.”

#### Analysis of Validity and Reliability

In this study, the internal consistency of the test scale was confirmed by Cronbach's α, and composite reliability (CR) was conducted to retest the reliability. Among the standards that have been commonly used in the past the Cronbach's α value and CR value should be >0.70 so that it is regarded as the acceptable standard, and the Cronbach's α value and CR value in this study were between 0.93 and 0.96, which meets the suggested standard, as shown in Table 1.

**Table 1:** Analysis of validity and reliability.

**Construct**	**α**	**CR**	**AVE**	**FL**	***FL***	***t***
Threshold	>0.70	>0.70	>0.50	>0.50	>0.50	>3
Short video flow	0.96	0.96	0.79	0.86	0.73–0.89	35.76–38.25
Short video addiction	0.93	0.93	0.68	0.83	0.71–0.92	26.92–30.85
Intrinsic learning motivation	0.96	0.96	0.84	0.91	0.87–0.94	34.59–43.57
Extrinsic learning motivation	0.95	0.95	0.78	0.89	0.87–0.91	38.69–44.22
Learning well-being	0.96	0.95	0.75	0.86	0.80–0.93	20.28–30.21

*α, Cronbach's α; FL, factor loadings; CR, composite reliability; AVE, average variance extraction*.

In this study, average variance extracted (AVE) and factor loading (FL) were used to determine the convergent validity. In confirmatory research, the FL value should be higher than 0.50 and if it is lower than this value the items should be deleted, while all the questions retained in this study met the criteria suggested by the scholars, where the mean FL values of the configurations ranged from 0.71 to 0.93, as shown in Table 1. In addition, when AVE value should be >0.50 to represent the stringent validity of the structure, and the AVE values ranged from 0.68 to 0.84, as shown in Table 1.

The confirmatory indicated that each construct has construct discriminant validity if its AVE root number value is larger than the Pearson correlation coefficient value of other constructs. The results of the analysis indicated that each of the constructs in this study had discriminant validity, as shown in Table 2.

**Table 2:** Discrimination validity analysis.

**Constructs**	**1**	**2**	**3**	**4**	**5**
1. Short video flow	(0.89)				
2. Short video addiction	0.74	(0.82)			
3. Intrinsic learning motivation	−0.38	−0.45	(0.92)		
4. Extrinsic learning motivation	−0.35	−0.42	0.84	(0.88)	
5. Learning well-being	−0.25	−0.33	0.70	0.64	(0.86)

*The value of AVE root number of the structure is in parentheses, and the other values are Pearson's correlation coefficients*.

### Data Analysis

The goal of social sciences is not only to perform basic statistical descriptions and identify individual factors and behaviors (related to specific social situations), but also to identify causal relationships between scientific domains of interest (i.e., variables). This requires complex statistical data analysis methods and techniques due to the complexity of social reality, that is, the underlying characteristics of many social phenomena (45). Survey data in the field of social sciences are often analyzed using Structural Equation Modeling (SEM). Having enough number of participants, SEM allows scholars to effortlessly establish and reliably test hypothetical associations between theoretical constructs and between constructs and their observed indicators (46). Therefore, this study used SPSS 23.0 for demographic variable analysis, descriptive statistics of the constructs, and reliability and validity analysis, in addition to AMOS 20.0 for item analysis, model fit analysis, and structural equation model validation.

## Results

### First-Order Confirmatory Analysis

First-order confirmatory factor analysis (First-order CFA) was conducted to confirm the fit of a single dimension; the statistical threshold value of χ^2^/*df* should be smaller than 5; the root mean square error of approximation (RMSEA) should be smaller than 0.10; goodness-of-fit index (GFI) and adjusted goodness-of-fit index (AGFI) should be >0.80; and items with factor loadings (FL) not higher than 0.50 must be removed from the original questionnaire. As shown in Table 3, the results of the deletion of questions were: Short video flow was reduced from eight to six items; short video addiction from eight to six; intrinsic learning motivation from six to five; extrinsic learning motivation from six to five; and learning well-being from 10 to 7.

**Table 3:** First-order CFA analysis.

**Index**	**χ^2^**	***df***	**χ^2^/** ***df***	**RMSEA**	**GFI**	**AGFI**
Threshold	–	–	<5	<0.10	>0.80	>0.80
Short video flow	23.30	9	2.59	0.06	0.99	0.97
Short video addiction	37.10	9	4.12	0.08	0.98	0.95
Intrinsic learning motivation	17.50	5	3.50	0.07	0.99	0.96
Extrinsic learning motivation	22.50	5	4.50	0.08	0.98	0.95
Learning well-being	62	14	4.43	0.08	0.96	0.93

### Descriptive Analysis

The descriptive analysis results of the construct showed that the *M* value of short video flow was 2.30, *SD* value was 1.16; the *M* value of short video addiction was 2.18, *SD* value was 1.13; the *M* value of intrinsic learning motivation was 3.65, *SD* value was 0.95; the *M* value of extrinsic learning motivation is 3.77, *SD* value is 0.92; the *M* value of learning well-being was 3.47, *SD* value was.98 as shown in Table 4.

**Table 4:** Descriptive analysis.

**Construct**	***M***	***SD***
Short video flow	2.30	1.16
Short video addiction	2.18	1.13
Intrinsic learning motivation	3.65	0.95
Extrinsic learning motivation	3.77	0.92
Learning well-being	3.47	0.98

*M, mean; SD, standard deviation*.

### Model Fit Analysis

Before conducting model verification in this study, model fit analysis was used to confirm the degree of fit of the research model. The recommended value of χ^2^/*df* for the proposed indicators should be <5; RMSEA values should be <0.10; GFI, AGFI, normed fit index (NFI), non-normed fit index (NNFI), comparative fit index (CFI), incremental fit index (IFI), and relative fit index (RFI) values should be larger than 0.80 (47); and parsimony normed fit index (PNFI) and parsimony goodness of fit index PGFI values should be >0.50. The fit index values for this study were χ^2^ = 1,314.1, *df* = 372, χ^2^/*df* = 3.53, RMSEA = 0.07, GFI = 0.87, AGFI = 0.85, NFI = 0.92, NNFI = 0.94, CFI = 0.94, IFI = 0.94, RFI = 0.92, PNFI = 0.85, and PGFI = 0.75.

### Path Analysis

**Figure 2:**
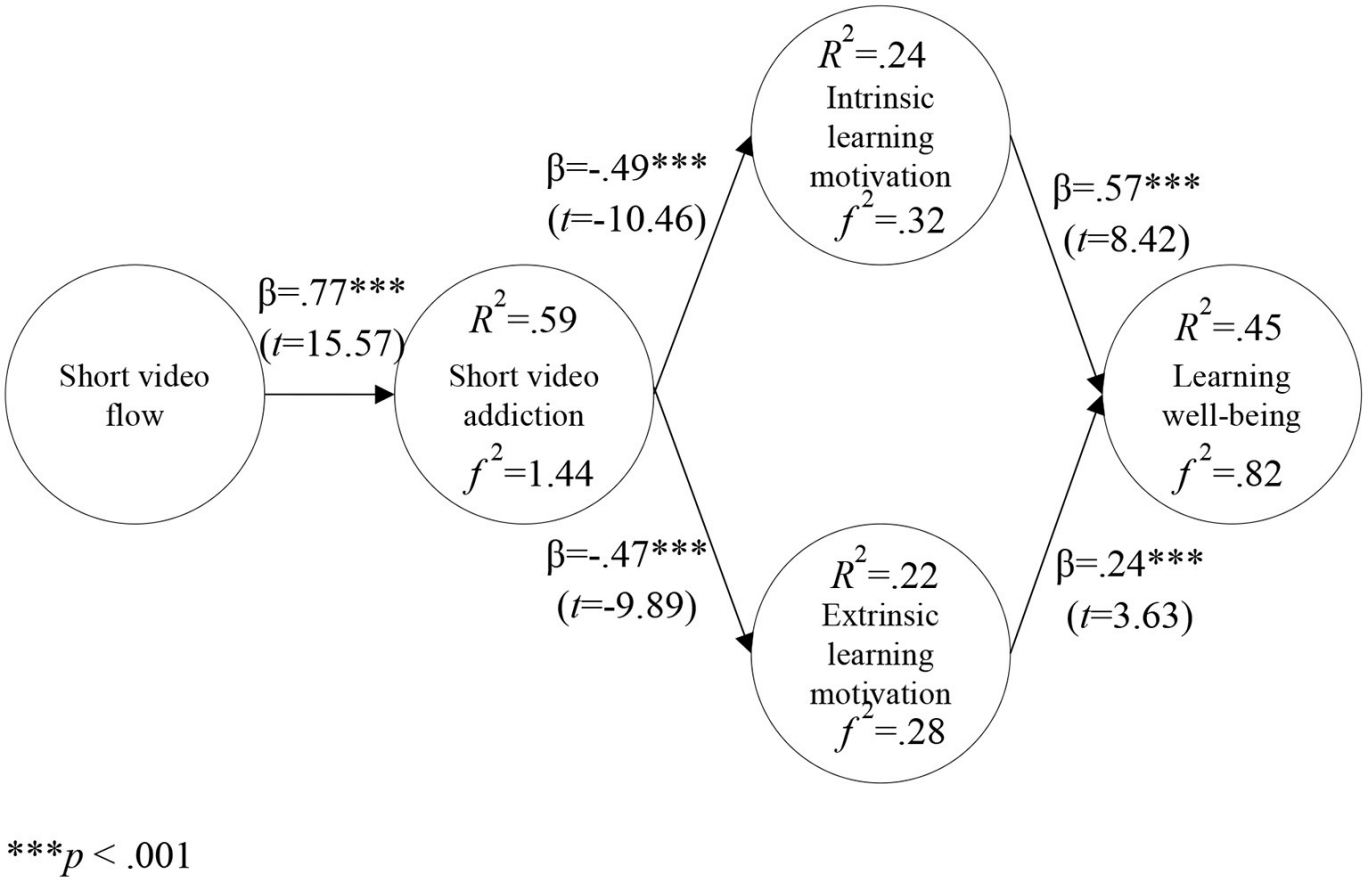
Model Verification. ***p <0.001.

This study proposes five research hypotheses based on the theory of flow and ecosystem theory and constructed a theoretical model, and validates it through a structural equation modeling. Model verification results showed that short video flow had a positive effect on short video addiction (β = 0.77^***^; *t* = 15.57); short video addiction had a negative effect on intrinsic learning motivation (β = −0.49^***^; *t* = −10.46); short video addiction had a negative effect on extrinsic learning motivation (β = −0.47^***^; *t* = −9.89); intrinsic learning motivation had a positive effect on learning well-being (β = 0.57^***^; *t* = 8.42); and extrinsic learning motivation had a positive effect on learning well-being (β = 0.24^***^; *t* = 3.63), as shown in Figure 2.

The explanatory power of short video flow for short video addiction was 59% with an *f2* of 1.44; the explanatory power of short-video addiction for intrinsic learning motivation was 24% with an *f2* of 0.32; the explanatory power of short video addiction on extrinsic learning motivation was 22% with an *f2* of 0.28; and the explanatory power of intrinsic and extrinsic learning motivation on learning well-being was 45% with an *f2* of 0.82, as shown in Figure 2.

### Indirect Effects Analysis

In this study, Bootstrapping was used to find the confidence interval of the indirect effect and to test the hypothesis of the mediated effect simultaneously. The results of the indirect effects analysis showed that short video flow had an indirect negative effect on intrinsic learning motivation (β = −0.38^**^); short video flow had an indirect negative effect on extrinsic learning motivation (β = −0.36^**^); short video flow had an indirect negative effect on learning well-being (β = −0.30^**^); and short video addiction had an indirect negative effect on learning well-being (β = −0.40^**^), as shown in Table 5.

**Table 5:** Indirect effect analysis.

**Construct**	**Short video flow**		**Short video addiction**	
	**β **	**95% CI**	**β**	**95% CI**
Intrinsic learning motivation	−0.38**	[−0.46, −0.28]		
Extrinsic learning motivation	−0.36**	[−0.45, −0.27]		
Learning well-being	−0.30**	[−0.38, −0.22]	−0.40**	[−0.49, −0.30]

**p < 0.01.

Good quantitative science emphasizes the use of the confidence interval (CI). The confidence limit of CI does not cross 0 to represent the statistical significance of the results of the validation analysis, meaning that the upper and lower bound values, whether positive or negative, should be in the same direction. As shown in Table 5, the 95% CI of the indirect effect analysis, the upper and lower bounds of this study are in the same direction and do not cross 0. This indicates that the results of the validation analysis in this study are statistically significant.

### Discussion

Flow experience is defined as an optimal experience in which an individual is completely immersed in an activity due to being energized, attentive, and successful during the activity (38), while the participants in this study perceived lower levels of short video flow (*M* = 2.30, *SD* = 1.16). Media addiction, as a mental disorder and a complex biopsychosocial phenomenon, is defined as a user's inability to control media use and its adverse effects on the user's daily life (3). Therefore, in this study, extending from the definition of media addiction, short video addiction was defined as an addictive behavior in which users use short video software excessively, inappropriately or dependently; participants in this study perceived low levels of short video addiction (*M* = 2.18, *SD* = 1.13). Intrinsic motivation refers to people's spontaneous curiosity and interest, their tendency to pursue challenges, and to train and advance their knowledge and skills to engage in tasks even in the absence of actionable rewards (48); participants in this study perceived moderate levels of intrinsic motivation to learn (*M* = 3.65, *SD* = 0.95). Extrinsic motivation comes from the external goals of the activity itself and involves being driven by externally imposed incentives and punishments to make oneself willing to participate in the task (49); participants in this study perceived moderate levels of extrinsic learning motivation (*M* = 3.77, *SD* = 0.92). Well-being is a core expressive indicator of optimal human functioning and is a state experienced by a person that emphasizes material success, comfort, and pleasure as a subjective feeling and an individual's awareness of inner satisfaction and pleasure (50, 51); participants in this study perceived moderate levels of well-being (*M* = 3.47, *SD* = 0.98).

The reason for the low overall mean of short video flow and short video addiction presented in this study can be explained by the argument of Kassas and Palma (52), who suggested that this result is due to the non-predisposed bias of the respondents in self-reporting, which is mainly a matter of personal seeking and social norms and perceptions, so that they would choose options that are more in line with the general public when filling out the survey. Although there may be biased responses in this study, since this was a correlation study, not a descriptive statistical study, which aimed to investigate the correlation between paths (13), it is evident from the validation results that the hypothetical paths between constructs are still valid.

#### Short Video Flow Has a Positive Effect on Short Video Addiction

People with high flow are fully immersed in the process of activity that they are doing (15). With the popularity of short video, flow experience has been commonly used to understand how much individuals immerse themselves in or interact with technology (16). The results of this study showed that short video flow has a positive effect on short video addiction which is consistent with previous study findings that individuals' flow experience is related to media addiction (17, 18). If learners experience a highly enjoyable short-video flow experience, it will increase the chance of learners' addictive behavior of using short videos.

#### Short Video Addiction Has a Negative Effect on Intrinsic and Extrinsic Learning Motivation

The results of this study showed that short video addiction had a negative effect on intrinsic and extrinsic motivation to learn. This result signifies that short video addiction had a negative influence on their daily life (20, 21), such as motivation to learn. Most studies indicated that addiction has more of a negative than a positive effect on emotional reactions, mood, and psychological problems (22, 23). In recent year, one study also pointed out that addiction can cause negative learning motivations in children (24), which is consistent with the results found in this study. That is, high levels of video addiction will have negative impacts on students' learning motivation. If learners perceive that they have a higher level of short video addiction, this will reduce their intrinsic and extrinsic motivation for the learning task.

#### Intrinsic and Extrinsic Learning Motivation Has a Positive Effect on Learning Well-Being

Several studies have indicated that motivation is an important factor for students' learning success rate (25) and is an important indicator to determine whether students have the ability to cope in that environment (26). Motivation also refers to the amount of information students absorb from the learning materials presented to them or how much they learn in a learning activity (27). Studies have suggested that motivation is an important determinant of student well-being (27, 29, 30), which is consistent with the result of this study which found that intrinsic and extrinsic learning motivation had positive effects on learning well-being. Therefore, this implies that when learners have a higher level of motivation to learn, it helps to increase their subjective well-being during the learning process.

#### Short Video Flow Has an Indirect Negative Effect on Intrinsic and Extrinsic Learning Motivation

How short video flow affects students' learning motivation may be a critical reason to failure in academic. The results of the present study showed that short video flow had an indirect negative effect on intrinsic and extrinsic learning motivation. However, when in flow, people are highly focused and concentrate on what they are doing at a given moment (33). In line with this, if learners are in a state of high enjoyment when watching short videos, they will become addicted to them, which will have a negative impact on their learning motivation.

#### Short Video Flow and Short Video Addiction Have an Indirect Negative Effect on Learning Well-Being

The results of this study indicated that short video flow had a negative effect on well-being, which indicates that the learning well-being of these participants who are highly enjoying short video flow experiences (34) is negatively impacted. This result is consistent with Kwok et al. (53) that excessive participation in social media may be problematic and may have a negative impact on the physical and mental health of young people. Furthermore, Laffan et al.'s (35) online gaming study; they found a negative and weak relationship between online game flow and overall well-being. Several studies have found that addiction may negatively affect users' wellbeing and that the subjective well-being of individuals who use online activities intensely may be affected (7, 37). Internet addiction is often challenged as a factor that influences teenagers' psychological health (1). The results of this study showed that short video flow and short video addiction had indirect negative effects on learning well-being. This indicates that if learners are in a state of high enjoyment when watching short videos and have short video addiction behaviors, it will negatively affect their subjective well-being in the learning process through learning motivation.

Chen et al. (54) and Chen et al. (55) indicated that past studies have found a significant positive relationship between problematic Internet use and psychological distress. That is, excessive use of the Internet hinders the development of positive psychology. In the more recent study, Chen et al. (56) found that problematic gaming, problematic social media use, and problematic smartphone use are important mediators of the association between psychological distress and increased time spent on Internet-related activities during the COVID-19 outbreak. This suggests that initial levels of issue-specific use in social media are associated with increased psychological distress (57). Therefore, failure to monitor or limit Internet behavior can have unintended negative consequences (58).

## Conclusion and Recommendation

### Research Limitations and Future Study

Garaigordobil (42) suggested that few studies have examined the correlation between age and well-being, and the existing findings are not very consistent. Although this study investigated the perceived learning well-being of vocational school students at the higher education level, it was not possible to confirm whether there were differential situations in the perceived learning well-being of learners at different ages within the same educational system. Therefore, in the follow-up study, in addition to comparing the perceived levels of learning well-being among elementary, junior high, high school, and university students, we can also explore their level of addiction to short videos.

In addition, Leung and Chen (3) suggest that qualitative research may help paint a picture of addiction, identify the process of addiction, elucidate addicts' perspectives, respond to questions, and explore how cultural factors influence perceptions of media. In this study, although it has been verified that flow experience has a positive effect on short video addictive behavior, and short video addictive behavior does negatively affect learning motivation, it was not possible to understand more deeply what is the meaning and value of short videos to students with addictive tendencies, and why the addictive behavior affects their learning. In addition, while demographics have also been used as an important variable to examine behavioral addiction in previous studies, the focus of this study is on theoretical model verification to examine the relationship between variables. It is therefore recommended that future studies may consider analyzing different demographic and may compare the results by gender, school, city, financial income, as well as other individual differences such as personal identities, skills and personality (59).

## Conclusion

This study aims to understand the causes of short video addiction and its impact on the psychology of learning, and to develop a research model that combines the perspectives of mind flow theory and ecosystem theory to investigate the relationship between short video flow, short video addiction, intrinsic and extrinsic learning motivation, and learning well-being. The results of the study showed that: (1) short video flow has a positive effect on short video addiction; (2) short video addiction has a negative effect on intrinsic and extrinsic learning motivation; (3) intrinsic and extrinsic learning motivation have positive effects on learning well-being; (4) short video flow has an indirect negative effect on intrinsic and extrinsic learning motivation; and (5) short video flow and short video addiction have indirect negative effects on learning well-being.

In addition, although the participants in this study reported low levels of addictive behaviors, the descriptive analysis showed that most used short videos frequently and for a long period of time, which may be due to the idea that they would choose to conform to social norms, as suggested by scholars, but also due to a cognitive gap and that they did not consider their use behavior as an addiction. Therefore, it is also recommended that parents, college teachers, and counselors can communicate to students the concept of behavioral addiction and the possible harm that short videos can cause to learning and mental health.

### Recommendation

From the above results, it is clear that addiction to short videos has a negative impact on learners' motivation and positive psychology of learning, but according to the software characteristics of short videos, it is inevitable that users will have a high level of flow experience, so it is difficult to eliminate this enjoyment experience. Therefore, according to the viewpoint of PPC theory, parents, college teachers, and counselors should cultivate college students' self-monitoring ability, effectively guide them to use short-video apps in a self-controlled manner, and watch short videos for a reasonable amount of time, so as to enhance their intrinsic and extrinsic learning motivation, and further enable them to feel a positive subjective sense of well-being in their learning experience, and at the same time reduce their dependence on short videos.

In addition, due to the negative impact of the addiction on students, this study also suggests that governments should set appropriate regulations on the use of online media for different student groups. For example, for underage students, they should be restricted from logging in and using their short video accounts late at night to ensure that they get enough rest time. For adult students, it is recommended to use the short video time every day as a reminder, and the short video software will be temporarily stopped when the accumulation exceeds a certain time on the same day to avoid excessive use of it. At the same time, the government can also sponsor schools and educational organizations to promote education on proper Internet use so that students can understand the dangers of problematic Internet use.

## Data Availability Statement

The raw data supporting the conclusions of this article will be made available by the authors, without undue reservation.

## Ethics Statement

Ethical review and approval was not required for the study on human participants in accordance with the local legislation and institutional requirements. Written informed consent for participation was not required for this study in accordance with the national legislation and the institutional requirements.

## Author Contributions

J-HY, Y-TW, Y-FW, and M-YC: concept and design and drafting of the manuscript. J-HY, Y-TW, Y-FW, and M-YC: acquisition of data and statistical analysis. M-YC and J-NY: critical revision of the manuscript. All authors contributed to the article and approved the submitted version.

## Conflict of Interest

The authors declare that the research was conducted in the absence of any commercial or financial relationships that could be construed as a potential conflict of interest.

## Publisher's Note

All claims expressed in this article are solely those of the authors and do not necessarily represent those of their affiliated organizations, or those of the publisher, the editors and the reviewers. Any product that may be evaluated in this article, or claim that may be made by its manufacturer, is not guaranteed or endorsed by the publisher.
